# Adipose-Derived Mesenchymal Stem Cells Reprogram M1 Macrophage Metabolism *via* PHD2/HIF-1α Pathway in Colitis Mice

**DOI:** 10.3389/fimmu.2022.859806

**Published:** 2022-06-10

**Authors:** Yin Yuan, Shuo Ni, Aoxiang Zhuge, Lanjuan Li, Bo Li

**Affiliations:** ^1^State Key Laboratory for Diagnosis and Treatment of Infectious Diseases, The First Affiliated Hospital, School of Medicine, Zhejiang University, Hangzhou, China; ^2^Department of Orthopedic Surgery and Shanghai Institute of Microsurgery on Extremities, Shanghai Jiaotong University Affiliated Sixth People’s Hospital, Shanghai, China; ^3^Jinan Microecological Biomedicine Shandong Laboratory, Jinan, China

**Keywords:** mesenchymal stem cells, colitis, macrophage polarization, glycolysis, HIF-1α

## Abstract

Ulcerative colitis (UC) is a chronic inflammatory bowel disease worldwide. Infiltration of pro-inflammatory macrophages (M1 macrophages) contributes to the occurrence of bowel inflammation. Transplantation of mesenchymal stem cells (MSCs) is a promising therapeutic strategy for UC, but the exact mechanism remains unknow yet. Here, we treated DSS-induced colitis mice with adipose‐derived mesenchymal stem cells (ADMSCs) and revealed that ADMSCs alleviated colon inflammation by reducing the infiltration of M1 macrophages. Moreover, ADMSCs exerted this therapeutic effect by inhibiting succinate accumulation, increasing PHD2 to prevent M1 macrophages from overexpressing HIF-1α and thereby reprogramming the glycolytic pathway of M1 macrophages. Meanwhile, the succinate secreted by M1 macrophages triggered ADMSCs to secrete PGE2 in return, which could also shift macrophages from M1 phenotype to M2. Our work demonstrated an immunomodulatory effect of ADMSCs and provided a novel perspective on UC therapy.

**Graphical Abstract:**
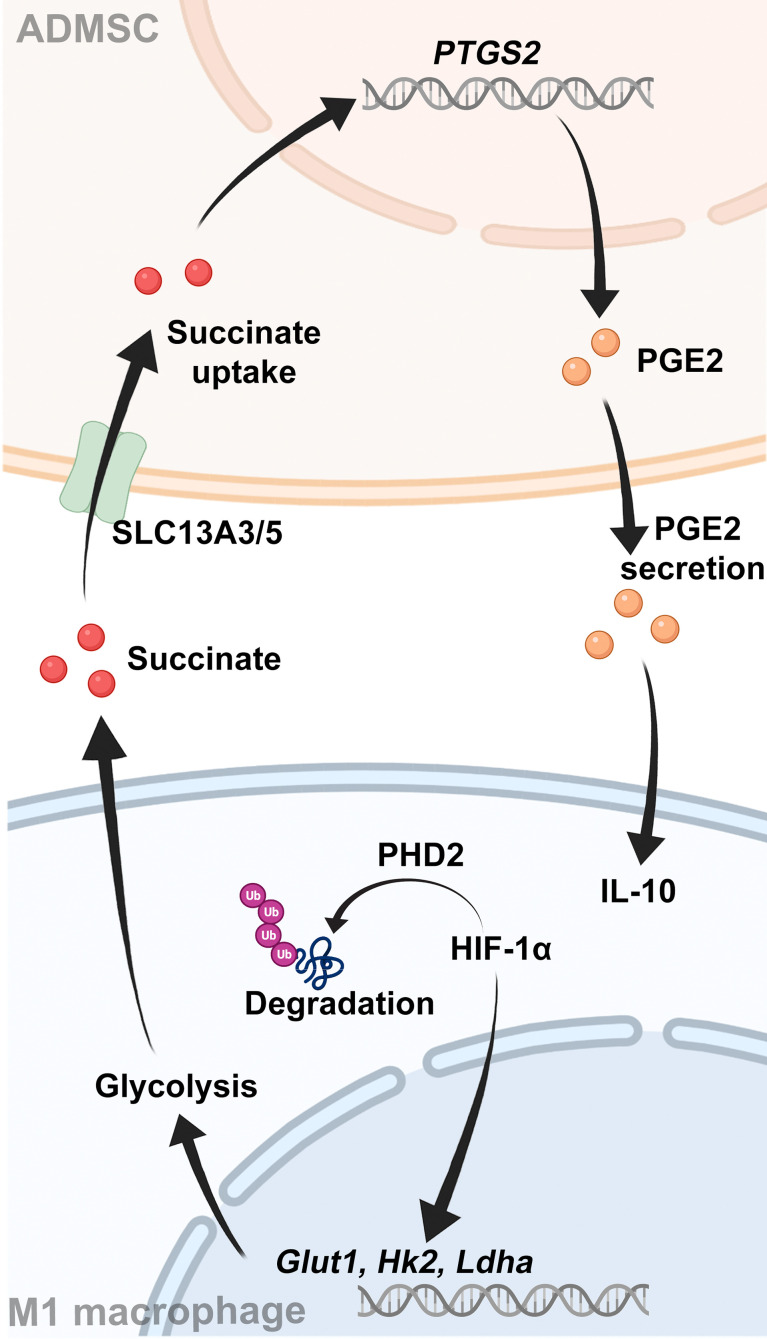
Schematic showing the interaction between ADMSCs and M1-macrophages.

## Introduction

Ulcerative colitis (UC) is a type of chronic inflammatory bowel disease (IBD) worldwide, characterizing by superficially mucosal inflammation limited in the rectum and the colon (1, 2). The exact pathogenesis of UC remains unknown yet but is thought to involve genetic predisposition, immune response and environmental triggers, such as diets, luminal microbes and antibiotics (3). Since most chronic inflammation is a consequence of dysregulated immune reaction, explorations on immune abnormalities have become a hot topic in UC (4, 5). At present, immunomodulators including infliximab, adalimumab and vedolizumab mainly target at specific immune factors to suppress the dysregulated immune system in UC patients (6, 7). However, these drugs are far from optimal for they are only effective in a very small subset, with many potential side effects such as infection and tumor (8).

Intestinal macrophages are important in establishing and maintaining gut immune homeostasis (9). Macrophages could secrete various cytokines and soluble factors to protect the host from affection in normal intestinal tracts (10). However, Different microenvironment results in heterogeneous activation phenotypes of macrophages, where their functions are also different (11). There are two major phenotypes of activated macrophages, the classically activated macrophages (M1 macrophages) and the alternatively activated macrophages (M2 macrophages). M1 macrophages exert a pro-inflammatory function for they could secrete various pro-inflammatory cytokines, chemokines and inflammatory mediators. Whereas, M2 macrophages are mainly involved in anti-inflammatory responses and tissues repairation (12). Several studies have revealed that the percentage of M1 macrophages increased in the colon tissues of UC patients and of animal models with colitis (13, 14). Upon inflammation environment, those macrophages were activated to certain phenotypes that aggravated bowel inflammatory reactions and accelerated the progress of UC (15).

Cellular metabolism alters dramatically along with the activation of macrophages (16). Glycolysis is a primary energy event of M1 macrophages, where glucose was converted to pyruvate, accompanied with a production of ATP (17). Excess glycolysis results in accumulation of glycolytic metabolites, such as itaconate, succinate and fumarate, to escape from mitochondria and exert pro-inflammatory effects (18). Inhibition of glycolysis and its metabolites could suppress macrophage M1 polarization, regulate inflammation response and restore immune homeostasis (19). Hypoxia inducible factor-1α (HIF-1α) is a pivotal factor that determines whether glucose is consumed through oxidative phosphorylation (OXPHOS) or glycolysis (20). However, under normoxic conditions, HIF-1α is rapidly hydroxylated and then degraded by prolyl hydroxylase domain 2 (PHD2) and the hydroxylase activity of PHD2 is modulated by various factors such as hypoxia and certain molecules (21). These evidences provide us with novel perspectives on UC pathogenesis and treatment.

Transplantation of mesenchymal stem cells (MSCs) is a safe and promising cell-based therapeutic strategy for UC (22, 23). After being transplanted to the host, MSCs could repair the damaged cells *in situ* by homing to the injured tissues and could also restore immune homeostasis by secreting anti-inflammatory cytokines and chemokines (24). However, approaches to acquire MSCs are quite limited and invasive (25, 26). Adipose‐derived mesenchymal stem cells (ADMSCs) are a type of MSCs isolated from adipose tissues. Subcutaneous white adipose tissues from the abdomen, thigh and hip were the most common tissue sources processed for ADMSCs isolation (27). Comparing to other kinds of MSCs, ADMSCs had the advantages of great accessibility and large abundance of tissue sources (28). Several studies have revealed that ADMSCs could alleviate peptic ulcer and acute injury by exerting anti-apoptosis and anti-inflammatory effects (29, 30). However, whether ADMSCs could treat UC and the potential mechanisms, remains unknown.

In this study, we found that transplantation of ADMSCs transplantation alleviated the DSS-induced colon inflammation and inhibited M1 macrophages infiltration. In details, ADMSCs exerted this inhibitory effect by reducing succinate accumulation and increasing PHD2 to prevent M1 macrophages from overexpressing HIF-1α, thereby reprogramming the glycolytic pathway of M1 macrophages. Meanwhile, the succinate secreted by M1 macrophages triggered ADMSCs to secrete PGE2 in return, which could further shift macrophages from M1 phenotype to M2. Our results provided a novel perspective on UC therapy.

## Materials and Methods

### Cells and Reagents

ADMSCs were purchased from the Innovative Precision Medicine Group (Hangzhou, China) and were cultured with α-MEM medium (Gibco, Gaithersburg, MD, USA) containing 15% FBS (Gibco). Bone marrow-derived macrophage cells (BMDMs) were isolated from C57BL/6 mice according to protocols. Briefly, cells were flushed out from mice femoral tissues. Then red blood cells were lysed and BMDMs were collected.

### Flow Cytometry

The surface markers of ADMSCs were assessed by flow cytometry. Briefly, 5 × 10^5^ ADMSCs were collected and resuspended with FACS buffer before they were incubated with specific antibodies in the dark for 30 minutes. Then all cells were washed with stain buffer twice and assessed by a Coulter flow cytometer (FC500MCL, Brea, CA). Information about PE-conjugated CD90, CD73, CD105, CD34, CD45 and CD117 antibodies were listed in the **Supplementary Table 1**. Results were analyzed by FlowJo Software (Ashland, OR).

### Co-Culture of ADMSCs and BMDMs

Transwell chambers with 0.4 μm pores (Corning, California, USA) were used in the co-culture experiments of ADMSCs and BMDMs. Before co-cultured with ADMSCs, BMDMs were polarized to M1 phenotype with the stimulation of 100 ng/mL LPS (MCE, Monmouth Junction, NJ, USA) and 20 ng/mL hIFN-γ (MCE) for 48 hours. Then ADMSCs and BMDMs were planted onto transwell inserts at the ratio of 1:1 and co-cultured for 24 hours before assessment.

### Extracellular Flux (XF) Assays

For XF assays, BMDMs were planted on XF24-well seahorse plates at the density of 5 × 10^4^ cells per well and stimulated with 100 ng/mL LPS and 20 ng/mL hIFN-γ for 48 hours. Then BMDMs were co-cultured with the same amount of ADMSCs using transwell chambers. After 24 hours, all chambers were removed and cell medium was replaced by XF medium. The OCR and ECAR values were assessed by an XF24 flux analyzer in accordance to the protocols. Results were normalized by cell numbers.

### Transfection

For transfection experiments, ADMSCs were transfected with siRNAs targeting at HIF-1α (Ribobio, Guangzhou, China) and Lipofectamine 3000 (Invitrogen, Carlsbad, CA, USA) in accordance to the manufacturer’s instructions. The sequences of siRNAs were listed in the **Supplementary Table 1**.

### DSS-Induced Colitis and ADMSCs Treatment *In Vivo*

All animal experiments obeyed the guidelines of the Animal Experimental Ethical Inspection of the First Affiliated Hosipital, Zhejiang University School of Medicine (2021-216). Briefly, 6-week-old male C57BL/6 mice were divided into three groups randomly: the control group, the DSS group and the ADMSCs+DSS group. Mice in the DSS and ADMSCs+DSS groups were provided with 3% DSS (MP Biomedicals, Santa Ana, CA, USA) in drinking water to develop colitis while mice in the control group were provided with regular drinking water for 7 days. 5 ×10^6^ ADMSCs resuspended in 250 μL PBS [human equivalent dose: 16 ×10^6^/kg (31)] were intraperitoneally injected to mice in MSC+DSS group on day 4 while equivalent PBS vehicle were injected to mice in the control and DSS groups on the same day. Weight loss, fecal occult blood and disease activity index (DAI) were monitored every day. On day 10, all mice were sacrificed and tissues were harvested for further assessments.

### Immunofluorescence Staining

For immunofluorescence staining, colon tissues were fixed in 4% paraformaldehyde overnight. Then fixed tissues were stained with anti-F4/80 antibody (#30325, CST, Beverly, MA, USA) and anti-inducible NO synthase (iNOS) antibody (#13120, CST). Images were photographed by a confocal microscope (Zeiss).

### Quantitative Real-Time PCR

RNA was extracted from cells with RNeasy Mini kits (Qiagen, Valencia, USA) and reverse-transcribed into cDNA with PrimeScript RT reagent kits (Takara, Kusatsu, Japan). The relative expression of mRNA was assessed by SYBR Green probes. mRNA expression was calculated by the Delta-Delta (ΔΔ) Ct method and all results were normalized to controls. Primers are listed in the **Supplementary Table 1**.

### Western Blotting

Proteins were extracted from cells with RIPA Buffers (Beyotime, Shanghai, China) containing protease inhibitor cocktails (Beyotime). Then proteins were separated by SDS-PAGE and were transferred to PVDF membranes for detection. Specific primary and secondary antibodies used in this study were listed in the **Supplementary Table 1**.

### Statistical Analysis

All data were presented as mean ± SD. Two-tailed t-test and one way ANOVA were used to assess the difference between groups. P value < 0.05 was considered as statistically significant.

## Results

### Characterization of ADMSCs

**Figure 1:**
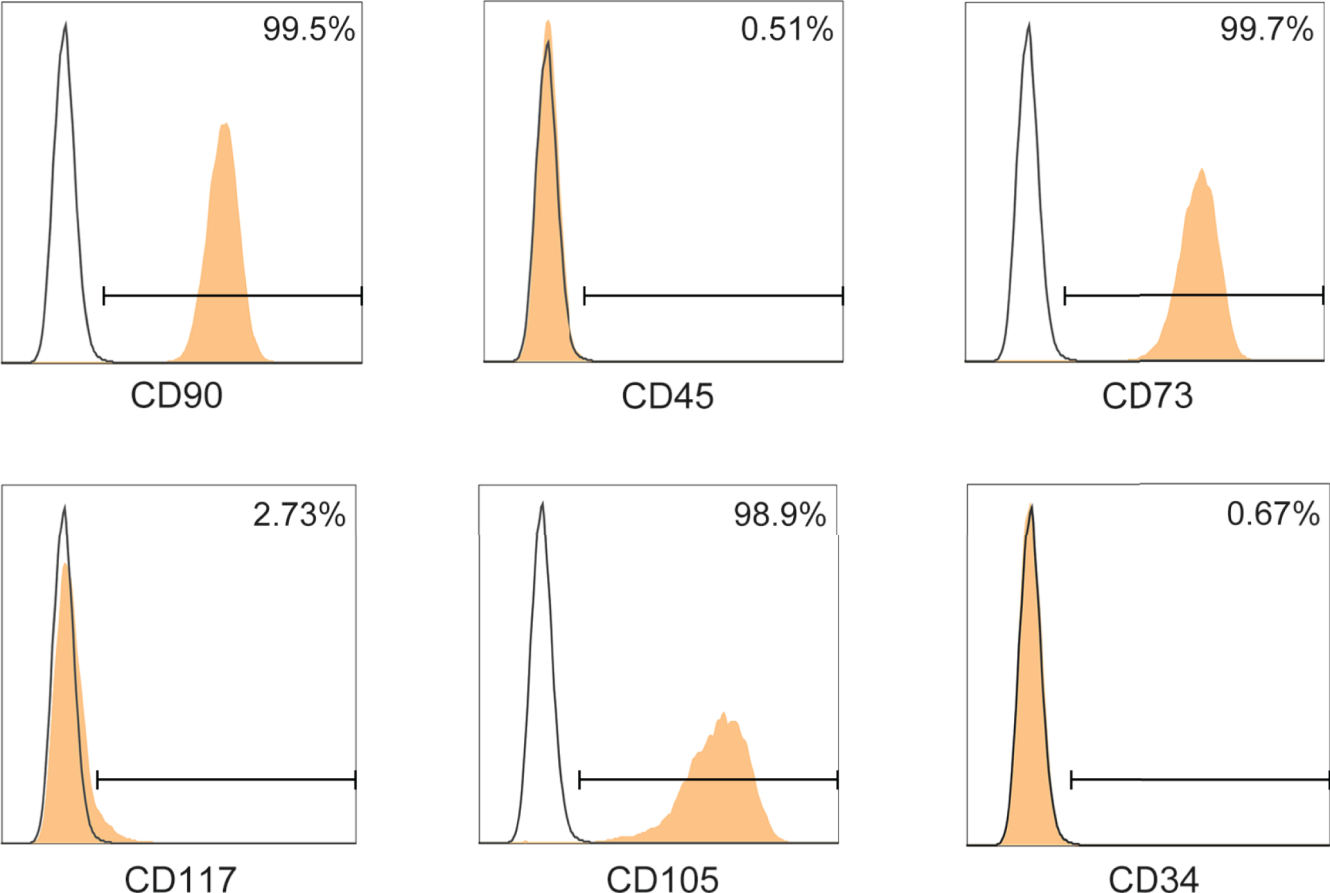
Phenotypic characterization of ADMSCs. Surface determinants of ADMSCs assessed by flow cytometer. Black lines represented the isotype control.

Firstly, we assessed the immunophenotype of ADMSCs according to the MSC Characteristics and Identification Criteria (32). MSCs biomarkers including CD90, CD73 and CD105 were positive in ADMSCs, while the leukocyte markers CD45, CD117 and CD34 were negative (**Figure 1**).

### ADMSCs Transplantation Ameliorated DSS-Induced Colitis and Inhibited M1 Macrophage Polarization

**Figure 2:**
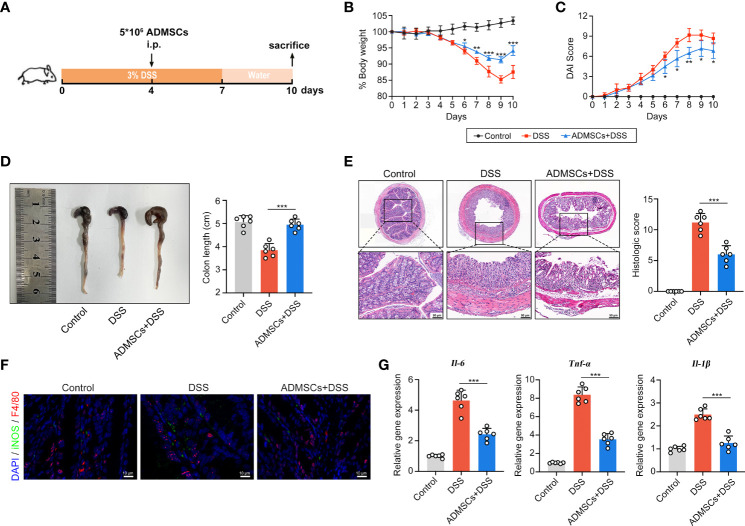
ADMSCs ameliorated DSS-induced colitis and inhibited M1 macrophages infiltration. **(A)** Schematic diagram of animal experiments. Mice in the DSS and ADMSCs+DSS groups were provided with 3% DSS in drinking water to develop colitis. 5 ×10^6^ ADMSCs were intraperitoneally injected to mice in MSC+DSS group on day 4. **(B–D)** Representative pictures and quantifications of bodyweight **(B)**, DAI scores **(C)** and colon lengths **(D)** from different groups. **(E)** H&E staining and histological scores of colon tissues from mice with different treatment. **(F)** Representative images of M1 macrophages infiltration in colon tissues assessed by Immunofluorescence staining. (green for F4/80, red for iNOS and blue for DAPI). **(G)** Gene expression of IL-6, TNF-α and IL-1β assessed by qRT-PCR. (*p < 0.05; **p < 0.01; ***p < 0.001).

To explore whether ADMSCs could ameliorate UC severity, DSS-induced colitis mouse models were intraperitoneally injected with ADMSCs (**Figure 2A**). Drinking DSS aqueous solutions induced a continuous reduction in mice bodyweight, while this DSS-induced weight loss was significantly alleviated by ADMSCs transplantation (**Figure 2B**). Additionally, the severity index of colitis measured by DAI scores was also decreased by ADMSCs transplantation (**Figure 2C**). Another typical morphological alteration in colitis mice is reductions in colon length (33). In this work, we found that the colon lengths of mice in the ADMSCs +DSS group were approximated to those in the control group, while mice in the DSS group exhibited significant reductions in colon length (**Figure 2D**). Furthermore, histopathological staining and analysis were also conducted in order to evaluate the severity of colitis more precisely (34). Comparing to the DSS group, colon tissues from mice in the ADMSCs+DSS group counted up less histologic scores for less mucosal erosion, crypt destruction and infiltration of inflammatory cell, suggesting a significant remission of colon inflammation upon ADMSCs treatment (**Figure 2E**). Given that the occurrence of bowel inflammation required M1 macrophages serving as antigen presenting cells, we further investigated the percentage of M1 macrophages (F4/80^+^iNOS^+^) by immunofluorescence staining. Notably, comparing to mice in the control group, the percentage of colonic F4/80^+^iNOS^+^ macrophages significantly increased in DSS-treated mice, while the percentage decreased with transplantation of ADMSCs (**Figure 2F**). Meanwhile, pro-inflammatory cytokines secreted by M1 macrophages also reduced upon ADMSCs treatment (**Figure 2G**). All of these results revealed that ADMSCs could ameliorate DSS-induced colon inflammation and inhibited M1 macrophage polarization in colitis mice.

### ADMSCs Inhibited M1 Polarization by Reprograming the Glycolytic Pathway in Macrophages

**Figure 3:**
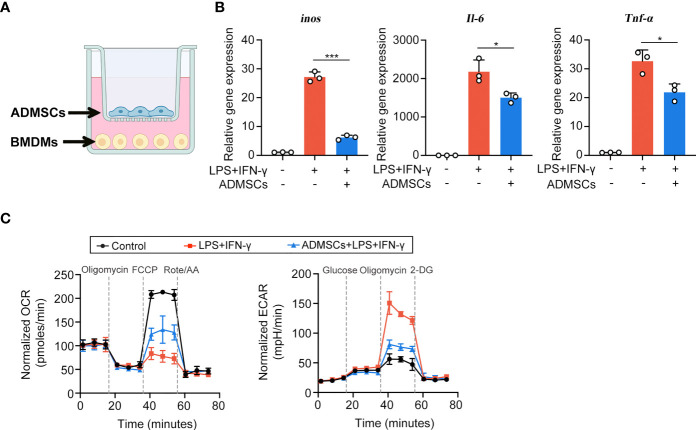
ADMSCs inhibited the M1 polarization and glycolytic pathway in macrophages. **(A)** Schematic diagram of the co-culture system. **(B)** qRT-PCR analysis of M1-characterized genes including iNOS, IL-6 and TNF-α. **(C)** Oxygen consumption rate (OCR) and extracellular acidification rate (ECAR) values of BMDMs with different treatments. (*p < 0.05; ***p < 0.001).

Then we explored the potential mechanisms through which ADMSCs exerted their inhibitory effects on M1 macrophages, using an *in vitro* system that co-cultured ADMSCs and bone-marrow-derived macrophages (BMDMs) (**Figure 3A**). BMDMs were polarized to M1 phenotypes with LPS and IFN-γ stimulation before co-cultured with ADMSCs. Consistent with the results of experiments *in vivo*, the gene expression of M1-characterized pro-inflammatory cytokines iNOS, IL-6 and TNF-α reduced significantly when BMDMs were co-cultured with ADMSCs, confirming that ADMSCs could also inhibit M1 polarization *in vitro* (**Figure 3B**). M1 macrophages exerts an enhanced glycolysis in order to meet their ATP requirements (20). Therefore, we assessed the OCR and ECAR values of BMDMs, as the readouts of their OXPHOS and glycolysis. Interestingly, comparing to M1-polarized BMDMs, significant increase of OCR and reduction of ECAR were found in those BMDMs co-cultured with ADMSCs, indicating that ADMSCs could constrain the glycolytic pathway in BMDMs, reducing intracellular ATP production, and thus inhibited M1 polarization (**Figure 3C**).

### ADMSCs Reprogrammed the Glycolysis Through Succinate-Dependent PHD2/HIF-1α Pathway in M1 Macrophages

**Figure 4:**
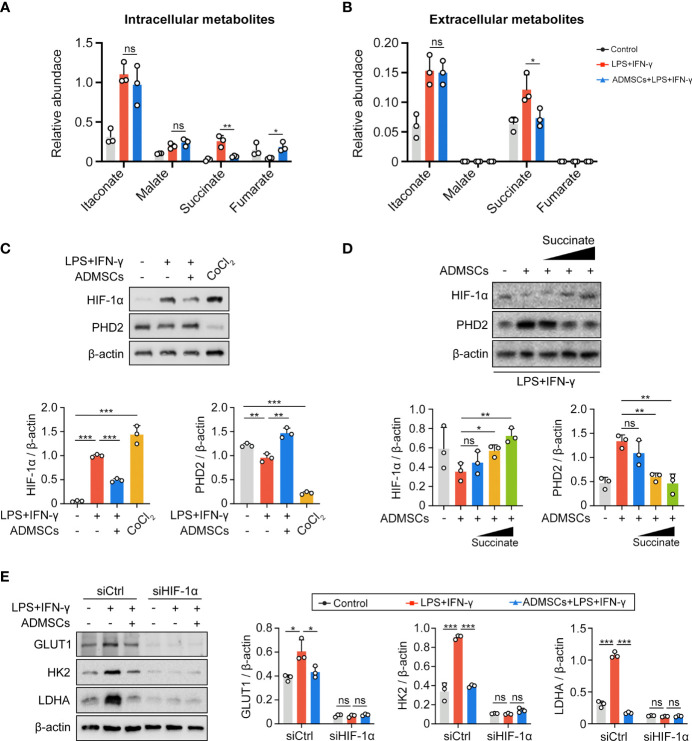
ADMSCs reprogrammed glycolysis in M1-BMDMs through succinate-dependent PHD2/HIF-1α pathway. **(A, B)** Intracellular **(A)** and extracellular **(B)** metabolites of BMDMs with different treatment. **(C)** HIF-1α and PHD2 levels in BMDMs with different treatments. **(D)** Protein levels of HIF-1α and PHD2 in BMDMs supplemented with ADMSCs and different concentrations of succinate. **(E)** Protein levels of glycolytic enzymes (GLUT1, HK2 and LDHA) in BMDMs transfected with HIF-1α siRNA. (*p < 0.05; **p < 0.01; ***p < 0.001; ns, no significance).

To further investigated the potential mechanism, we assessed both intracellular and extracellular metabolite contents of BMDMs. Both intracellular and extracellular itaconate and succinate were increased along with the enhancement of glycolysis in M1-BMDMs, but only succinate decreased upon ADMSCs treatment, while itaconate remained unchanged (**Figures 4A, B**). Therefore, we speculated that ADMSCs reprogrammed the glycolysis of M1-BMDMs by inhibiting both intracellular and extracellular succinate accumulation. HIF-1α is a key factor to determine whether glucose is consumed through OXPHOS or glycolysis (21). Interestingly, HIF-1α levels increased in M1-BMDMs but decreased when M1-BMDMs were co-cultured with ADMSCs (**Figure 4C**). Furthermore, we found that co-culture with ADMSCs could increase the level of PHD2, who could mediate the protein degradation of HIF-1α, and supplementation of succinate could decrease PHD-2 levels (**Figure 4D**). Glycolytic enzymes including GLUT1, HK2, LDHA, etc. are very important in glycolysis because they can facilitate rapid glucose uptake and transformation intracellularly (35). Interestingly, we found that ADMSCs reduced the levels of either GLUT1, HK2 and LDHA in M1-polarized BMDMs, and these inhibitory effects of ADMSCs on the glycolytic enzymes were eliminated when HIF-1α was silenced by short interfering RNA (siRNA) (**Figure 4E**). Together, all these results demonstrated that ADMSCs reprogrammed the glycolytic pathway of M1 macrophages *via* succinate-dependent PHD2/HIF-1α pathway.

### Macrophage-Derived Succinate Triggered ADMSCs to Uptake Succinate in Return and Secrete PGE2 to Shift Macrophages From M1 Phenotype to M2

**Figure 5:**
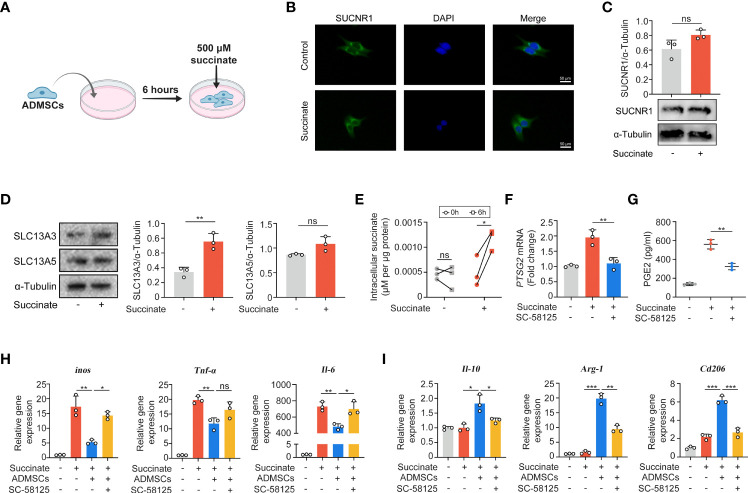
Macrophage-derived succinate triggered ADMSCs to uptake succinate and secreted PGE2 to further shift macrophages from M1 phenotype to M2. **(A)** Schematic diagram of ADMSCs exposed to succinate *in vitro*. **(B, C)** Representative microscopy images **(B)** and western blot analysis **(C)** of SUCNR1 in ADMSCs. **(D)** Protein levels of SLC13A3 and SLC13A5 in ADMSCs supplemented with or without succinate. **(E)** Concentrations of intracellular succinate in ADMSCs. **(F, G)** Ptsg2 gene expression **(F)** and PGE2 concentrations **(G)** in ADMSCs. **(H, I)** Gene expression of M1-characterized cytokines **(H)** and M2-characterized cytokines **(I)** in BMDMs. (*p < 0.05; **p < 0.01; ***p < 0.001; ns, no significance).

Given that succinate is an important intercellular signal molecule, we further explored whether succinate could regulate physiological processes of ADMSCs in return. The major receptor for succinate, succinate receptor 1 (SUCNR1), had no statistical alteration when ADMSCs were exposed to succinate *in vitro* (**Figures 5A–C**). However, levels of SLC13A3 and SLC13A5, two dicarboxylate co-transporters involved in succinate transport, increased significantly when ADMSCs were treated with succinate, consistent with an increasement of intracellular succinate concentrations (**Figures 5D, E**). Prostaglandin E2 (PGE2) is a major immune regulator secreted by ADMSCs (36). We measured PGE2 concentrations in culture media from ADMSCs and the gene expression of prostaglandin-endoperoxide synthase 2 (Ptgs2). Both PGE2 concentrations and the gene expression of Ptgs2 in ADMSCs increased upon succinate treatment, and pre-stimulation with SC-58125 (Ptgs2 blocker) rescued this succinate-induced increasement (**Figures 5F, G**). Then we further investigated the effect of PGE2 on macrophage polarization. Notably, those ADMSCs pre-treated with succinate exerted a reduction in the gene expression of M1-characterized cytokines (iNOS, IL-6 and TNF-α) and an increasement of M2-characterized cytokines (IL-10, Arg-1 and CD206), while all these alterations were rescued by SC-58125 (**Figure 5H**). These results revealed that the PGE2 secreted by ADMSCs could shift M1 macrophages toward an M2 phenotype.

## Discussion

Stem cell is a novel and promising strategy for UC therapy. However, the application potentials of stem cells were quite limited for their poor source and invasive approaches. In this work, we used a kind of accessible and continuous MSCs isolated from adipose tissues to investigate their therapeutic effects on UC. Previous studies have reported that MSCs could regulated macrophages polarization through cell-to-cell contact, secretion of soluble factors, extracellular vesicles and mitochondrial transfer (37). However, the exact mechanism of how ADMSCs regulated M1 macrophages in UC patients remains unknow. In this study, we found that transplantation of ADMSCs could ameliorate DSS-induced colitis and reduced M1 macrophages infiltration by reprogramming their glycolytic pathway. M1-macrophages is characterized by an enhanced intracellular glycolysis (18). We found that transplantation of ADMSCs increased PHD-2 levels and leaded to an enhanced HIF-1α degradation in macrophages by inhibiting the accumulation of intracellular succinate, a kind of glycolysis metabolite. HIF-1α is an important transcription factor of glycolytic enzymes, and the reduction of HIF-1α resulted in decreases of those glycolytic enzymes, which then limited the production of ATPs and thus inhibited M1 polarization.

Several studies have revealed that the therapeutic potentials of MSCs were mainly attributed to soluble factors and extracellular vesicles (38, 39). Interestingly, we found that those succinates secreted by M1 macrophages triggered succinate uptake by ADMSCs in return, resulting in increased intracellular succinate levels. Accumulation of intracellular succinate further stimulated ADMSCs themselves to secrete soluble molecule PGE2, which then exerted an anti-inflammatory effect on macrophages and shift macrophages from M1 phenotype to M2.

However, succinate receptor SUCNR1 is an early detector to many physiological and pathological processes, including obesity, inflammation and cancer (40, 41). Our results showed that there was no significant difference in SUCNR1 upon ADMSCs treatment. We speculated that the SUCNR1 signaling pathways in colitis mice might was activated by an enhancement of SUCNR1 protein activity rather than its protein level, as was reported by Gilissen et al. (42). Therefore, more studies are still needed to further investigate the mechanism in SUCNR1 signaling.

## Conclusion

ADMSCs transplantation inhibited the M1 polarization of macrophages by reprogramming their metabolism processes. Meanwhile, the metabolites secreted by M1 macrophages triggered ADMSCs to secrete anti-inflammatory cytokines in return. Our results provided a novel perspective on UC therapy and interaction between MSCs and macrophages.

## Data Availability Statement

The original contributions presented in the study are included in the article/supplementary files. Further inquiries can be directed to the corresponding authors.

## Ethics Statement

All animal experiments obeyed the guidelines of the Animal Experimental Ethical Inspection of the First Affiliated Hosipital, Zhejiang University School of Medicine (2021-421).

## Author Contributions

YY and SN drafted and revised the manuscript. AZ performed formal analysis. BL and LL provided project supervision, administration and funding. All authors contributed to the article and approved the submitted version.

## Funding

This work was supported by the National Key Science and Technology Project of China (2018YFC2000500), Research Project of Jinan Microecological Biomedicine Shandong Laboratory (JNL-2022051B), the Independent Task of State Key Laboratory for Diagnosis and Treatment of Infectious Diseases (2022zz22) and the National Natural Science Foundation of China (81790631).

## Conflict of Interest

The authors declare that the research was conducted in the absence of any commercial or financial relationships that could be construed as a potential conflict of interest.

## Publisher’s Note

All claims expressed in this article are solely those of the authors and do not necessarily represent those of their affiliated organizations, or those of the publisher, the editors and the reviewers. Any product that may be evaluated in this article, or claim that may be made by its manufacturer, is not guaranteed or endorsed by the publisher.

## Supplementary Material

Supplementary Table 1 Primers and antibodies used in this work.
