# Supplementary material for: Adipose-Derived Mesenchymal Stem Cells Reprogram M1 Macrophage Metabolism via PHD2/HIF-1α Pathway in Colitis Mice
Source: Front Immunol. 2022 Jun 10;13:859806. doi: 10.3389/fimmu.2022.859806 (PMC9226317; doi:10.3389/fimmu.2022.859806)
Supplement: Supplementary Table 1 — Primers and antibodies used in this work. [file Table_1.docx]

**Supplementary Table 1. Primers and antibodies used in this work.**

**Primers for qRT-PCR.**

| **Genes** | **Forward (5′-3′)** | **Reverse (5′-3′)** |
| --- | --- | --- |
| **TNF-α** | CATCTTCTCAAAATTCGAGTGACAA | TGGGAGTAGACAAGGTACAACCC |
| **IL-6** | GAGGATACCACTCCCAACAGACC | AAGTGCATCATCGTTGTTCATACA |
| **IL-1β** | CAACCAACAAGTGATATTCTCCATG | GATCCACACTCTCCAGCTGCA |
| **Arg1** | TGGCTTGCGAGACGTAGAC | GCTCAGGTGAATCGGCCTTTT |
| **IL-10** | CGGTTAGCAGTATGTTGTCCAGC | CGGGAAGACAATAACTGCACCC |
| **iNOS** | CTGATGGCAGACTACAAAGACG | TGGCGGAGAGCATTTTTGAC |
| **CD206** | CTACTGTTATGTCGCTGGCAAA | GGATGGAAGCAAAGTGGATTAG |
| **GAPDH** | ATCATCCCTGCATCCACT | TCTTCAGGGCTTTCTCGTTC |

**Sequences of siRNAs.**

| **Target genes** | **Sequences** |
| --- | --- |
| **siCtrl** | UAGCGACUAAACACAUCAA |
| **siHIF-1α #1** | CCUAUAUCCC AAUGGAUGAUGTT |
| **siHIF-1α #2** | TTGGAUAUAGGGUUACCUACUAC |

**Antibodies**

| **Antibody** | **Manufacturer** | **Cat. Num.** |
| --- | --- | --- |
| **PE-conjugated CD90** | BD Biosciences | 561970 |
| **PE-conjugated CD45** | BD Biosciences | 560975 |
| **PE-conjugated CD73** | BD Biosciences | 561014 |
| **PE-conjugated CD117** | BD Biosciences | 561682 |
| **PE-conjugated CD105** | BD Biosciences | 560839 |
| **PE-conjugated CD34** | BD Biosciences | 560941 |
| **HIF-1α** | CST | #36169 |
| **PHD2** | CST | #4835 |
| **β-actin** | Boster Biological Technology | BM0627 |
| **α-Tubulin** | Boster Biological Technology | BM1452 |
| **GLUT1** | CST | #73015 |
| **HK2** | CST | #2867 |
| **LDHA** | CST | #2012 |
| **SUCNR1** | Invitrogen | PA5-98179 |
| **SLC13A3** | Invitrogen | PA5-110375 |
| **SLC13A5** | Invitrogen | PA5-113058 |
